# Evaluation of frailty in geriatric patients undergoing cardiac rehabilitation after cardiac procedure: results of a prospective, cross-sectional study

**DOI:** 10.1186/s13102-024-00937-y

**Published:** 2024-07-02

**Authors:** Carolin Steinmetz, Laura Krause, Samra Sulejmanovic, Sabrina Kaumkötter, Johanneke Hartog, Bart Scheenstra, Flohr Stefan, Thomas Mengden, Clemens Grefe, Ernst Knoglinger, Nils Reiss, Birna Bjarnason-Wehrens, Thomas Schmidt, Monika Sadlonova, Christine A. F. von Arnim, Stephanie Heinemann

**Affiliations:** 1 Department of Geriatrics, University Medical Center Goettingen, Robert-Koch-Straße 40, 37075 Goettingen, Germany; 2 Department of Sports Science, University of Hildesheim, Universitätsplatz 1, 31141 Hildesheim, Germany; 3 Schüchtermann-Schiller’sche Clinic, Ulmenallee 11, 49214 Bad Rothenfelde, Germany; 4 Department of Cardio-Thoracic Surgery, University of Groningen, University Medical Center Groningen, Hanzeplein 1, 9713 GZ Groningen, Netherlands; 5 Department of Cardiothoracic Surgery, Heart and Vascular Center, Maastricht University Medical Center, Professor Debyelaan 25, 6229 HX Netherlands Maastricht,; 6 Department of Rehabilitation, Kerckhoff Heart Center, Ludwigstraße 41, 61231 Bad Nauheim, Germany; 7 Clinic and Rehabilitation Center Lippoldsberg, Birkenallee 1, 34399 Wesertal, Germany; 8 Kirchberg-Clinic Bad Lauterberg, Kirchberg 7-11, 37431 Bad Lauterberg, Germany; 9 Institute of Cardiology and Sports Medicine, Department Preventive and Rehabilitative Sport and Exercise Medicine, German Sport University, Am Sportpark Müngersdorf 6, 50933 Cologne, Germany; 10 Department of Psychosomatic Medicine and Psychotherapy, University of Goettingen Medical Center, Robert-Koch-Straße 40, 37075 Goettingen, Germany; 11 Department of Cardiovascular and Thoracic Surgery, University of Goettingen Medical Center, Robert-Koch-Straße 40, 37075 Goettingen, Germany; 12 German Center for Cardiovascular Research (DZHK), partner site Goettingen, Robert-Koch-Straße 42a, 37075 Goettingen, Germany

**Keywords:** Cardiac surgery, Valve intervention, Frailty, Cardiac rehabilitation

## Abstract

**Background:**

Frailty is an indicator of a decline in quality of life and functional capacity in cardiac rehabilitation (CR) patients. Currently, there is no standardized assessment tool for frailty used in CR. The aim of this study was to determine if the Clinical Frailty Scale (CFS) is feasible for assessing frailty in CR.

**Methods:**

Prospective, cross-sectional study within the framework of the ongoing multicenter prehabilitation study "PRECOVERY". Patients ≥75 years undergoing CR after cardiac procedure (n=122) were recruited in four German inpatient CR facilities. Assessments included: CFS, Katz-Index, hand grip strength (HGS), Short Physical Performance Battery (SPPB) and six-minute-walk test (6MWT). Outcomes were frailty (CFS≥4) and the correlation of frailty with assessments of functional capacity, activities of daily living and clinical parameters. Statistical analysis included descriptive statistics and correlations, using the spearman correlation coefficient and chi-square test to test for significance.

**Results:**

Data from 101 patients (79.9±4.0 years; 63% male) were analyzed. The mean CFS score was 3.2±1.4; 41.6% were defined as frail (CFS≥4). The mean time required to assess the CFS was 0.20 minutes. The findings show that CFS correlates significantly (p<0.001) with the following factors: Katz-Index, HGS, SPPB-Score and 6MWT (r≤-0.575). In addition, CFS correlated with small to moderate effects with co-morbidities (r=0.250), as-needed medications and need for nursing assistance (r≤0.248).

**Conclusions:**

The CFS assessment can be performed in under one minute and it correlates significantly with assessments of functional capacity, activities of daily living and clinical parameters in the CR setting.

**Trial registration:**

German Clinical Trials Register (DRKS; http:// www. drks. de; DRKS00032256). Retrospectively registered on 13 July 2023.

## Introduction

Due to demographic changes, the number of older people suffering a cardiovascular disease (CVD) is increasing significantly [1]. CVDs rarely occur alone, and their treatment requires careful consideration of various other age-related health problems (e.g., frailty, cognitive and sensory limitations) [2]. In studies with cardiac patients, the frailty status varies from 10% to 80% depending on the pathology, setting and frailty assessment tool used [3, 4]. In the cardiac rehabilitation (CR) setting, a short and efficient frailty assessment method is needed which correlates well with functional capacity due to the fact that frailty and functional capacity assessments can be time-consuming and strenuous for especially older CR patients with CVD.

One study reviewed the frailty-related research literature and identified 67 different frailty assessment tools [5]. In a review focused on the assessment of frailty in cardiac patients, up to 20 different frailty measurement tools were used [3]. All frailty assessments vary widely in terms of the number of variables and the amount of time required [6]. The reason for the numerous frailty assessment tools in different settings and cohorts is the lack of consensus on the definition of frailty and the lack of a gold standard assessment tool [5, 7–9].

The physical Frailty Phenotype [10] is the most commonly used frailty assessment tool in the research literature, followed by the Deficit Accumulation Index [5, 11, 12].

The Clinical Frailty Scale (CFS) is one of the top five highly-cited instruments used in frailty-related research literature [5]. The CFS is a multidimensional screening assessment based on a medical history and clinical examination that is frequently used in hospitals, especially in the setting of cardiology [6, 13]. Results of a longitudinal study in the inpatient setting of cardiology showed that the CFS predicts mortality and readmission as well as worsening disability after 1 year [14]. The significant advantage of the CFS lies in its simplicity and efficiency, enabling health staff (e.g., nurses) to assess frailty [15].

Currently, there is no standardized instrument for assessing frailty in the CR setting, but the need to assess frailty status is growing due to the increasing number of geriatric cardiac patients admitted to CR. In its "Call to Action" article, the European Association of Preventive Cardiology (EAPC) recommends the Edmonton Frail Scale [16] and CFS [17] as appropriate tools for the evaluation of frailty in the context of CR in order to better plan patients´ management [6]. The CFS could be an ideal measurement tool to assess frailty in the context of CR, as it is easy to administer and is associated with adverse events [14, 15].

To follow the call to action by the EAPC, we conducted a prospective cross-sectional study within the framework of the ongoing multicenter prehabilitation study "PRECOVERY" [21]. This is one of the first studies which can provide information about the evaluation of frailty using the CFS in the context of CR and how frailty status correlates to functional and clinical parameters in older CR participants.

The purpose of this study is to evaluate if the CFS is feasible to screen patients 75 years and older for frailty in the setting of CR after a cardiac procedure, and whether CFS scores correlate significantly with functional capacity, activities of daily living and clinical parameters (e.g., number of regular medications or need for nursing assistance). Furthermore, the CFS administration time is of interest. Based on standardized frailty assessment, additional assessments can be performed and specific, individualized programs can be tailored to benefit frail older CR patients.

## Methods

**Fig. 1:**
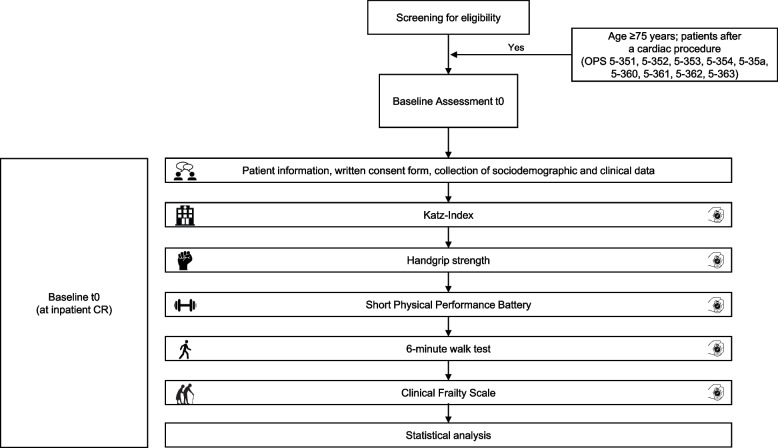
Research design and conducted assessments of the cross-sectional study. Abbreviations. *CR* cardiac rehabilitation, *OPS* operation and procedure codes

Patients who were ≥75 years old after cardiac procedure in four inpatient cardiac rehabilitation centers in Germany were asked to participate in this prospective, cross-sectional study. The assessments took place during inpatient CR (iCR) stay. Patients were not included if they were unable to give written consent due to poor German language skills or cognitive impairments. In addition, the amount of time needed to perform of each assessment was measured with a stopwatch (details see Fig. 1). The CFS was used to identify frail patients. Patients with a score ≥ 4 were considered to be frail [15, 17]. Further details about the patient population, the setting and assessments can be found in our recently published article [18].

The main research questions of this prospective, cross-sectional study are:

- Is the CFS feasible for assessing frailty in the CR setting?
- Is there a significant unconditional correlation between CFS and functional capacity and activities of daily living (measured with HGS, SPPB score, 6-minute walk distance [6MWD] and Katz-Index)?
- Is there a significant unconditional correlation between CFS and clinical parameters (number of co-morbidities, number of medications, need for nursing assistance and degree of disability)?
- Which of the performed assessments - CFS, HGS, SPPB, 6MWT and Katz-Index - requires the least amount of time to administer?

### Sample size calculation

When the sample size is 100, a one-sided 97.5% confidence interval for a correlation coefficient $\rho =0.5$(being the true underlying coefficient) will have a lower limit of 0.337. The sample size was determined based on the feasibility of the study and is not based on previous trials. The confidence intervals contained in Table 3 form the basis for calculating the sample size. This precision is sufficient to describe the population and base further research on the results.

### Sociodemographic data and medical history

The sociodemographic data includes age, sex, a statement about living alone, an officially recognized need for nursing assistance and any degree of disability recognized by the pension office. The parameter nursing assistance was assessed as an ordinal variable (level of care 1-3), whereas the degree of disability was assessed as a binominal variable. In Germany, the Medical Service carries out care assessments on the basis of SGB XI (Social Care Insurance) [25] for six modules using a standardized assessment tool (e.g., mobility, cognitive and communicative skills). Persons are classified either as needing no care or into one of the five levels of nursing assistance (1: Minor impairment of independence or abilities; 5: Severe impairment of independence or abilities with special requirements for nursing care) [25]. To be considered in need for nursing assistance, the need for care must exist for at least six months and be based on up to two diagnoses requiring care [25].

In Germany, a degree of disability indicates how severely a person is affected by their disability. The grade of disability can range from 20% to 100%. The higher the value, the more severe the disability. The degree of disability is usually awarded by physicians in local private practices who are commissioned by the pension office [26]. The physicians assess the degree of disability according to fixed rules. These rules are called medical care principles. People with a disability of 50% are considered severely disabled and therefore receive certain benefits (e.g., discounts on local public transport) [26].

The medical history consists of cardiac and non-cardiac concomitant diseases, cardiovascular procedure(s) with indication for CR and the number of “regular” medications and “as needed” medications.

### Functional capacity and frailty outcomes

Functional capacity, activity of daily living and frailty outcomes were assessed using handgrip strength (HGS), Short Physical Performance Battery (SPPB), and 6-minute walk test (MWT), Katz-Index and CFS. These outcomes were measured during the inpatient CR. The HGS was measured using a Jamar dynamometer (Jamar Hand Dynamometer, IL, USA). At the assessment of the HGS participants were asked to start with their dominant hand (right-handed or left-handed) followed by the non-dominant hand. Then, the test was repeated with the stronger hand. The second attempt with the stronger hand was defined as the maximal HGS. This approach varies from international protocols [19] and was modified by the study team due to logistical and practical reasons. All parameters, except CFS, were assessed by qualified, trained study personnel during the CR stay. The CFS assessment was performed by an independent physician who is familiar with that type of frailty assessment. The independent physician was not informed about the results of the other measurements performed in the cross-sectional study. With this separation of assessors, it was possible to ensure that the functional assessments did not influence the CFS results. Further details about the conducted assessments can be found in the previous published article [18].

### Statistical analysis

The Kolmogorov–Smirnov test was used for the assessment of normal distributions. Continuous data are presented as median and interquartile range (IQR) or mean ± standard deviation, while categorical data are presented as frequency and percentages. For the calculation of the bivariate correlation between the ordinal variable CFS and the interval variables HGS, SPPB and 6MWD, the spearman coefficient with one-way analysis of variance (ANOVA) was conducted. The same test was applied in the calculation of the correlation between CFS and the clinical parameters (number of co-morbidities, number of medication and officially recognized need for nursing assistance). To analyze the correlation between CFS and the nominal clinical parameter degree of disability the chi square test was used. All bivariate correlations were checked for the influence of the confounding variable “sex” by partial correlations [20]. In all analyses, a p<0.05 was considered statistically significant. All analyses except the bivariate and partial correlations were performed using IBM Statistical Package for the Social Sciences (SPSS) Version 21.0 (IBM Co., Armonk, NY, USA). The bivariate and partial correlations were calculated in R (Version 1.1, R Foundation for Statistical Computing, Vienna, Austria) [20].

## Results

### Study population

A total of 122 patients were screened for eligibility. Of these, 21 were excluded due to ineligible diagnoses (percutaneous coronary intervention, n=14; pacemaker/implantable cardioverter-defibrillator, n=4; others, n=3). A sample of 101 patients (79.7±4.0 years; 63% males) were included into the study.

The patient characteristics are presented in Table 1. Based on the CFS score, the participants were divided into two groups of frail and non-frail patients. During the inpatient CR stay, 41.6% of the cohort was diagnosed to be frail. The CFS was assessed in participants (*n*=101). The group of frail patients had significantly more often a chronic lung disease (e.g., COPD; *p*=0.030), chronic pain (*p*=0.016) or a kidney disease (*p*=0.029) in comparison to non-frail patients (CFS score < 4).

**Table 1:** Patient characteristics

Characteristics	All	frail patients (CFS ≥ 4)	non-frail patients (CFS < 4)	*P*-value
	(*n* = 101)	(*n* = 42)	(*n* = 59)	
	*(mean ± SD) or*	*(mean ± SD) or*	*(mean ± SD) or*	
	*(n, %)*	*(n, %)*	*(n, %)*	
Age (years)	79.7±4.0	80.3±4.4	79.3±3.7	^a^*p*=0.222
Sex				
male	64 (63%)	21 (50%)	43 (73%)	^**b**^***p*=0.019**
female	37 (37%)	21 (50%)	16 (27%)	
Number of medications (regularly)	9.0±3.0	9.1±3.0	9.0±3.1	^a^*p*=0.854
Number of medications (p.r.n.)	1.3±1.4	1.1±1.4	1.5±1.4	^a^*p*=0.169
Officially recognized need for nursing assistance	24 (24%)	13 (31%)	11 (19%)	^a^*p*=0.146
Any degree of disability recognized by the pension office	27 (27%)	12 (29%)	15 (25%)	^b^*p*=0.725
Living situation				
Living alone	27 (27%)	11 (26%)	16 (27%)	^b^*p*=0.917
Cardiac procedure^c^				
CABG-surgery	44 (33%)	16 (31%)	28 (34%)	^b^*p*=0.350
Valve surgery (e.g., mitral valve replacement)	50 (38%)	19 (37%)	31 (38%)	^b^*p*=0.871
Valve intervention (e.g., MitraClip, TAVI)	20 (15%)	11 (22%)	9 (11%)	^b^*p*=0.174
Concomitant diseases				
Coronary artery disease	73 (72%)	28 (67%)	45 (76%)	^b^*p*=0.288
Myocard infarction	21 (21%)	8 (19%)	13 (22%)	^b^*p*=0.715
Heart failure	75 (74%)	34 (81%)	41 (70%)	^b^*p*=0.194
Cardiac arrhythmias	64 (63%)	26 (62%)	38 (64%)	^b^*p*=0.797
Stroke	10 (10%)	3 (7%)	7 (12%)	^b^*p*=0.434
Peripheral artery disease	12 (12%)	5 (12%)	7 (12%)	^b^*p*=0.995
Hypertension	92 (91%)	37 (88%)	55 (93%)	^b^*p*=0.373
Diabetes mellitus	24 (24%)	11 (26%)	13 (22%)	^b^*p*=0.629
Asthma bronchiale	10 (10%)	7 (17%)	3 (5%)	^b^*p*=0.055
Chronic lung disease (e.g. COPD)	13 (13%)	9 (21%)	4 (7%)	^**b**^***p*=0.030**
Rheumatic disease	6 (6%)	2 (5%)	4 (7%)	^b^*p*=0.672
Arthrosis	34 (34%)	16 (38%)	18 (31%)	^b^*p*=0.426
Gout	15 (15%)	8 (19%)	7 (12%)	^b^*p*=0.317
History of fractures	51 (51%)	20 (48%)	31 (53%)	^b^*p*=0.626
Chronic pain	16 (16%)	11 (26%)	5 (9%)	^**b**^***p*=0.016**
Kidney disease	27 (27%)	16 (38%)	11 (19%)	^**b**^***p*=0.029**
Cancer (at any time)				
Breast	6 (6%)	2 (5%)	4 (7%)	^b^*p*=0.672
Prostate	12 (12%)	3 (7%)	9 (15%)	^b^*p*=0.214
Colon	3 (3%)	1 (2%)	2 (3%)	^b^*p*=0.768
Number of co-morbidities	5.6±1.9	5.9±1.6	5.4±2.1	^a^*p*=0.206

Abbreviations. *CFS* Clinical Frailty Scale, *SD* standard deviation, *n* number, *e.g.* for example, *p.r.n.* pro re nata, *TAVI* transcatheter aortic valve implantation, *COPD,* chronic obstructive pulmonary disease

^a^ Independent t-test

^b^ Chi-square-test

^*c*^ Multiple responses

### Outcomes

The results of the Kolmogorov–Smirnov test indicated that only HGS was normally distributed. The distribution of the other continuous variables was reviewed together with a biostatistician to assess the appropriateness of the test procedures used. The results of the Katz-Index, CFS, HGS, SPPB and 6MWT during inpatient CR are summarized in Table 2. It is important to note that these results show that frail patients performed significantly worse in all assessments compared to non-frail patients (p≤0.018). Compared to the Katz-Index, HGS, SPPB and 6MWT, the assessment of the CFS required the least amount of time (0.2±0.2 minutes).

**Table 2:** Overall results, and results differentiated and compared between frail and non-frail patients

Characeristics	Duration (min.)	All	frail patients	non-frail patients	*p*-value
		(*n* = 101)	(*n* = 42)	(*n* = 59)	(independent t-test)
	*mean±SD*	*mean±SD*	*mean±SD*	*mean*±*SD*	
		*median [IQR]*	*median [IQR]*	*median [IQR]*	
Katz-Index	0.5±0.3	5.7±0.9	5.4±1.1	5.9±0.6	***p*=0.018**
		6 [6-6]	6 [5-6]	6 [6-6]	
Handgrip strength (kg)	1.2±0.3	24.9±9.9	20.5±8.0	28.0±10.0	***p*<0.001**
		26 [18-32]	21 [15-26]	30 [21-35]	
SPPB score	3.7±1.7	7.5±3.3	6.2±3.5	8.5±2.7	***p*<0.001**
		8 [6-10]	6 [4-8]	9 [7-11]	
6MWD (meters)	> 6.0	288.8±136.5	208.5±124.4	345.9±115.0	***p*<0.001**
		315 [206-380]	210 [145-315]	360 [290-430]	
CFS	0.2±0.2	3.2±1.4	4.5±0.8	2.3±0.8	***p*<0.001**
		3 [2-4]	4 [4-5]	3 [2-3]	

Abbreviations. *min.* minute, *IQR* interquartile range, *SD* standard deviation, *CFS* Clinical Frailty Scale

**Fig. 2:**
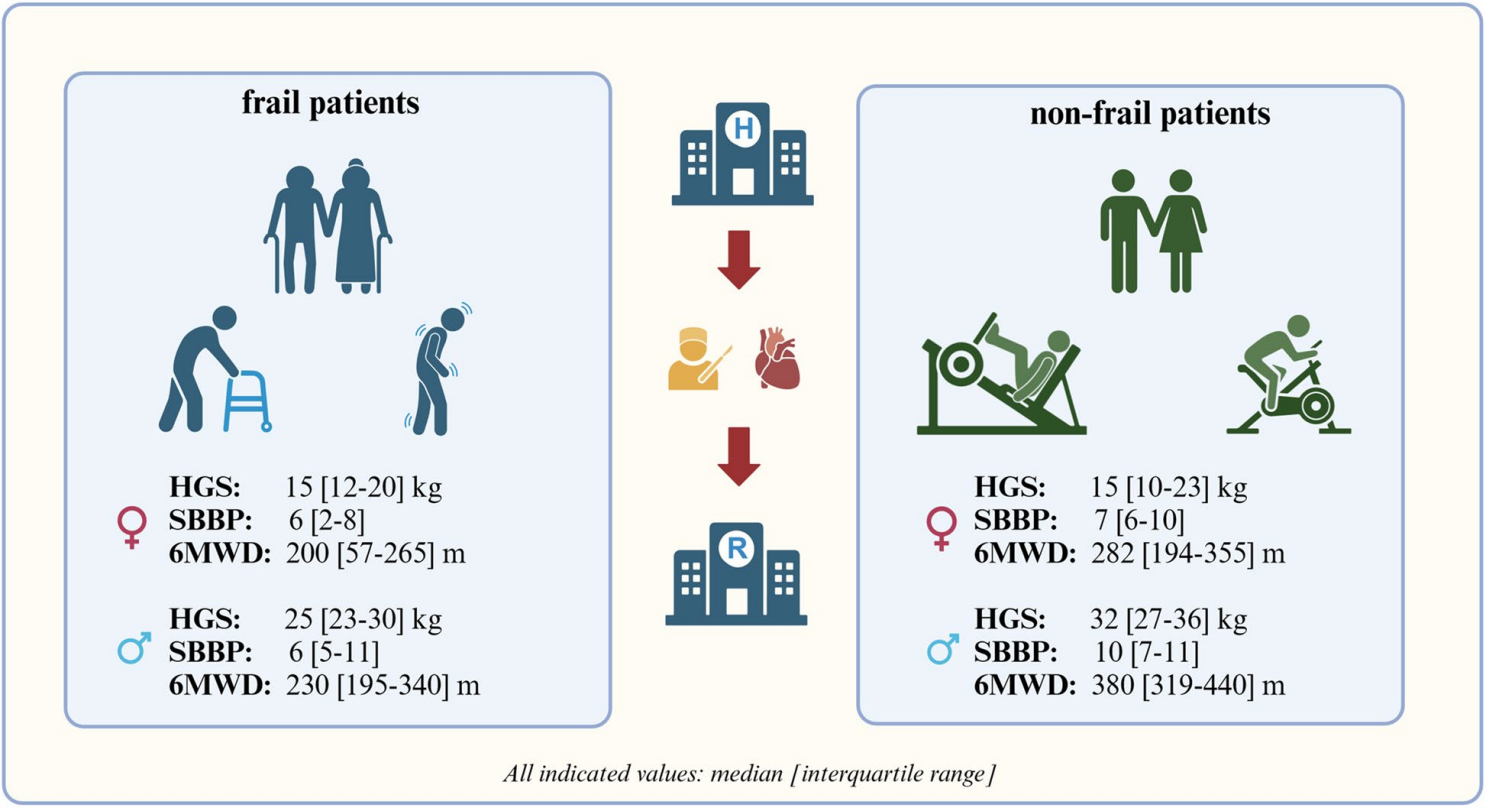
Illustration of functional capacity of frail and non-frail patients during inpatient CR after cardiac procedure (created with BioRender.com). Abbreviations. *HGS* hand grip strength, *kg* kilogram, *SPPB* Short Physical Performance Battery, *6MWD* 6-minute walk distance, *m* meter

Figure 2 illustrates the sex-specific functional capacity of frail and non-frail patients during iCR measured by HGS, SPPB and 6MWD. Differences in functional capacity are more pronounced for frail versus non-frail men than for frail versus non-frail women (see Fig. 2).

The calculation of the spearman correlation with one-way ANOVA revealed a significant correlation between frailty measured by the CFS and the Katz-Index (r= -0.326; *p*<0.001), 6MWD (r= -0.575*; *p*<0.001), SPPB score (r= -0.448; *p*<0.001) and HGS (r= -0.398; *p*<0.001). Likewise, we found significant bivariate correlations between CFS and the number of co-morbidities (r=0.250; *p*=0.012), the number of p.r.n. medications (r=-0.205; *p*=0.041) as well as the officially recognized need for nursing assistance (r=0.248; *p*=0.013) (see Table 3).

**Table 3:** Results of the correlations between CFS and functional capacity as well as clinical parameters

	CFS			
	correlation coefficient	sig. (2-sided)	95% CI	sample size
Katz-Index	r= -0.326^a^	*p*<0.001	-0.463 to-0.154	*n* = 101
HGS	r= -0.398^a^	*p*<0.001	-0.557 to-0.217	
SPPB Score	r= -0.448^a^	*p*<0.001	-0.607 to-0.252	
6MWD	r= -0.57^a^	*p*<0.001	-0.690 to -0.429	
Number of co-morbidities	r= 0.250^a^	*p*=0.012	0.062 to 0.410	
Number of medication [r.]	r= 0.139^a^	*p*=0.167	-0.045 to 0.318	
Number of medication [p.r.n.]	r= -0.20^a^	*p*=0.041	-0.394 to-0.006	
Level of care	r= 0.248^a^	*p*=0.013	0.063 to 0.401	
Degree of disability	N/A	*p*=0.994^b^	N/A	

Abbreviations. *CFS* Clinical Frailty Scale, CI Confidence interval (Bootstrap CI with 1000 bootstrap samples), *HGS* hand grip strength, *SPPB* Short Physical Performance Battery, *6MWD* six-minute-walk distance, *p.r.n.* pro re nata, *sig.* significant

^a^ Correlation coefficient calculated according to Spearman

^b^ Correlation coefficient calculated according to chi-square test

## Discussion

In this cross-sectional study, we evaluated if the CFS is feasible to screen patients ≥ 75 years for frailty in the setting of CR after a cardiac procedure, and whether the CFS scores correlate significantly with functional capacity, activities of daily living and clinical parameters (e.g., number of comorbidities, number of regular medications or need for nursing assistance). Furthermore, the administration time of the different assessments was of interest.

This is one of the first studies to measure frailty using the CFS during a CR inpatient stay and to correlate different physical and clinical parameters taking into account the confounding variable "sex".

Our results show 41.6% of the cohort to be frail at the entry of inpatient CR (CFS ≥ 4 points) following a cardiac procedure. Frail patients were more often diagnosed with chronic lung or kidney disease and chronic pain than non-frail patients. Frail patients are characterized by an increased vulnerability to acute and chronic diseases [21]. Especially older CVD patients often suffer from co-morbidities, as well as polypharmacy and reduced physical function [22]. Additionally, chronic lung and kidney diseases are based on age-related changes which can be further accelerated by the presence of clinical CVD [23].

In the setting of CR, frailty is often an unrecognized condition in patients with CVD. The assessment of frailty in CR in currently published studies ranges from the modified Fried Criteria focusing on functional cut-off values [24] to a Comprehensive Geriatric Assessment based on the Frailty Index (FI) by Rockwood [25] and FI independently [26, 27]. Manfredi et al. [28] measured frailty using a version operationalized by Survey of Health, Ageing and Retirement in Europe (SHARE) based on Fried’s five dimensions: “exhaustion”, “shrinking”, “weakness”, “slowness” and “low activity” [10]. All described methods vary widely, are time-consuming, resource-intensive and physically demanding for this multimorbid, older cohort. Such frailty assessments may be practical in study settings but not in the daily clinical routine of the CR setting. In contrast, the CFS required less than one minute to perform. In our cross-sectional study, CFS was easy to implement and feasible for assessing the frailty status of each participant. In none of the above-mentioned studies were the frailty results correlated with physical or other clinical outcomes. Instead, the focus was mainly on the development of frailty during the inpatient stay or the association between frailty level at CR and long-term outcomes [24–28]. In the studies of Südermann et al. [4, 29], frailty was assessed preoperatively in a cohort similar to ours. Correlations were performed between the frailty score and 30-day as well as 1-year mortality [4, 29]. Frailty was assessed by Comprehensive Assessment of Frailty (CAF) and Frailty predicts death One yeaR after CArdiac Surgery Test (FORECAST). In their article, the authors’ described that they performed the CFS but did not publish any results about it [4, 29]. So far, only one oncological study was found which measured frailty using the CFS and correlated these results with a performance status scale [30]. The target was to evaluate if the CFS is associated with prognosis and care needs at hospital discharge. The authors reported 81.8% of the patients to be frail and that the CFS correlated significantly with performance status measured by The Eastern Cooperative Oncology Group Performance Status Scale (ECOG PS) [30]. Frail patients were less likely to be discharged to their homes and a higher CFS value was significantly associated with a poorer prognosis [30]. In the field of oncology, the use of CFS is limited but recommended [30]. In their “Call to action“ Vigorito et al. [6] recommended CR clinicians to incorporate an easy-to-use tool based on a standardized subjective evaluation of frailty [6]. The CFS could be such an easy-to-use, standardized, subjective frailty assessment tool.

Approximately every second patient in our cohort exceeded the threshold for frailty. Frail patients performed significantly worse in all functional tests. Furthermore, frail patients had higher values in nearly all clinical parameters in comparison to non-frail patients. The sex-specific differences in the functional capacity of frail and non-frail patients are interesting: the differences are much more pronounced in men than in women. This is an indication that frailty has a greater influence on functional capacity in men compared to women. On the other hand, the lowest values for functional capacity were measured in frail women.

The measurement of HGS, SPPB-Score and 6MWT are frequently used tools to assess a patients´ functional capacity in the setting of CR. Compared to similar studies, our HGS values were much lower [24, 31]. This could be due to the older age and the different diagnoses in our cohort. We only included patients who recently underwent a cardiac procedure, whereas other studies included different diagnoses [24, 31]. This is an indication that in a cohort of older CVD patients, physical weakness is to be expected after a cardiac procedure and that particularly frail patients require measures to increase strength during CR. The SPPB scores in frail as well as non-frail patients assessed in our study are the lowest compared to the values described in other published articles [24, 25, 32]. Reasons for these differences may be the older age of our cohort and that our assessments took place mainly at the beginning/during CR and not at the end of CR, when patients are generally in much better condition. These results demonstrate that the SPPB is a good measurement to assess mobility in this cohort. The 6MWD assessed at CR varies widely between frail and non-frail participants. Due to the small number of frail patients in our study and the older age of our cohort, the 6MWD values are clearly below those described by Bencivenga et al. [25]. In contrast, Lutz et al. [24] showed similar results to our CR patients in a larger cohort [24, 25]. In comparison to these results, non-frail patients in all CR studies reached significantly longer 6MWD than frail patients. All non-frail patients in our cohort reached the cut-off value of 300 m [33]. A 6MWD under 300 meters is associated with high mortality risk and/or poor health status as well as reduced results in physical function tests (e.g., HGS, one-leg standing time) [33]. In conclusion, our results show that frail patients screened for frailty with the CFS perform significantly worse in the 6MWT compared to non-frail patients.

The assessment of frailty using the CFS in combination with selected functional assessments (e.g., SPPB, HGS) in the CR setting seems to be necessary to tailor rehabilitation programs for older and frail CR patients after a cardiac procedure. Studies show that especially resistance and balance training in addition to the regular CR program improves functional capacity at CR discharge in this frail cohort [34, 35]. Likewise, these kinds of CR programs have been able to reduce physical frailty levels in patients after valve surgery/intervention [35]. In the future, screening for frailty including functional assessments as well as tailored programs for frail individuals should be paired with the evaluation of outcomes in the CR setting in order to demonstrate the success of rehabilitation in this cohort.

### Strengths and limitations

A strength of this cross-sectional study is the focus on patients aged 75 years and older. Such older cardiac patients are becoming more common in CR but studies focusing on patients 75 and older have been relatively sparse. Furthermore, a sex-specific analysis of functional capacity in frail and non-frail patients in CR adds to the current knowledge about geriatric patients in this setting. The data were collected in four different inpatient CR facilities to increase the generalizability of the results. Previous studies in this field have collected data monocentrically in younger cohorts with different CR diagnoses mostly at CR admission and discharge.

There are some limitations to our study. This is a relatively small cohort study. Further studies with larger cohorts are needed to confirm the presented results. The average time measurement for HGS indicates that the study personnel did not always follow the international standards requiring one minute of rest between the attempts. Furthermore, a comparison of the results with a gold standard for assessing frailty (e.g. a Comprehensive Geriatric Assessment) would be desirable in order to emphasize the quality of the CFS. Another limitation is the cross-sectional design of the study, as data is only assessed at one time point. A 12-month follow-up after inpatient CR as a re-survey in the clinical setting with a repeat of all assessments would be desirable to examine the long-term effect of rehabilitation in frail and non-frail patients.

## Conclusions

Over 40% of older patients entering inpatient CR after a cardiac procedure are frail. Frailty status correlates significantly, as anticipated, with poor functional capacity, reduced activities of daily living and some clinical parameters (e.g., a higher number of co-morbidities, an officially recognized need for nursing assistance). The CFS is a simple clinical measure to screen patients for frailty after a cardiac procedure at CR entry, requiring less than one minute to administer. There is a large sex difference in functional capacity when comparing frail and non-frail patients. These results indicate that the assessment of frailty using CFS is feasible in the setting of CR. Our findings can be used to design other studies with similar geriatric cohorts, including the entire patient pathway from the clinical preoperative assessment, the procedure, CR and long-term post-procedural care programs.

## Data Availability

The anonymized raw dataset is available on request. Request should be addressed to the corresponding author: Carolin Steinmetz, carolin.steinmetz@med.uni-goettingen.de, +49-551-39-68248.

## Abbreviations

CABG: Coronary artery bypass graft surgery
CAF: Comprehensive Assessment of Frailty
CFS: Clinical Frailty Scale
CR: Cardiac rehabilitation
CVD: Cardiovascular diseases
DRKS: German Clinical Trials Register
e.g.: for example
FORECAST: Frailty predicts death One yeaR after Elective CArdiac Surgery Test
HGS: Handgrip strength
CR: Cardiac rehabilitation
iCR: Inpatient cardiac rehabilitation
m: meter
MCID: Minimal clinically important difference
min: Minutes
OPS: Operation and procedure codes catalogue
p.r.n.: pro re nata
SHARE: Survey of Health, Ageing and Retirement in Europe
SPPB: Short Physical Performance Battery
6MWD: 6-minute walk distance
6MWT: 6-minute walk test
